# The Long Intron 1 of *Growth Hormone* Gene from Reeves’ Turtle (*Chinemys reevesii*) Correlates with Negatively Regulated GH Expression in Four Cell Lines

**DOI:** 10.3390/ijms17040543

**Published:** 2016-04-12

**Authors:** Wen-Sheng Liu, Jing-E Ma, Wei-Xia Li, Jin-Ge Zhang, Juan Wang, Qing-Hua Nie, Feng-Fang Qiu, Mei-Xia Fang, Fang Zeng, Xing Wang, Xi-Ran Lin, Li Zhang, Shao-Hao Chen, Xi-Quan Zhang

**Affiliations:** 1 Department of Animal Genetics, Breeding and Reproduction, College of Animal Science, South China Agricultural University, Guangzhou 510642, China; magiel1018@stu.scau.edu.cn (J.-E.M.); lweixia_2001@163.com (W.-X.L.); zhjg-2005@163.com (J.-G.Z.); wangjuan901226@126.com (J.W.); nqinghua@scau.edu.cn (Q.-H.N.); qff_1804@163.com (F.-F.Q.); mxfang@jnu.edu.cn (M.-X.F.); zimo999888@163.com (F.Z.); wangxing635@163.com (X.W.); 15626195391@163.com (X.-R.L.); zhangli761101@163.com (L.Z.); 200930710106@stu.scau.edu.cn (S.-H.C.); 2 Guangdong Provincial Key Lab of Agro-Animal Genomics and Molecular Breeding, South China Agricultural University, Guangzhou 510642, China; 3 Key Lab of Chicken Genetics, Breeding and Reproduction, Ministry of Agriculture, South China Agricultural University, Guangzhou 510642, China; 4 College of Marine Sciences, South China Agricultural University, Guangzhou 510642, China

**Keywords:** turtle, pituitary gland, *growth hormone* gene, intron, expression

## Abstract

Turtles grow slowly and have a long lifespan. Ultrastructural studies of the pituitary gland in Reeves’ turtle (*Chinemys reevesii*) have revealed that the species possesses a higher nucleoplasmic ratio and fewer secretory granules in growth hormone (GH) cells than other animal species in summer and winter. *C. reevesii GH* gene was cloned and species-specific similarities and differences were investigated. The full *GH* gene sequence in *C. reevesii* contains 8517 base pairs (bp), comprising five exons and four introns. Intron 1 was found to be much longer in *C. reevesii* than in other species. The coding sequence (CDS) of the turtle’s *GH* gene, with and without the inclusion of intron 1, was transfected into four cell lines, including DF-1 chicken embryo fibroblasts, Chinese hamster ovary (CHO) cells, human embryonic kidney 293FT cells, and GH4C1 rat pituitary cells; the turtle growth hormone (tGH) gene mRNA and protein expression levels decreased significantly in the intron-containing CDS in these cell lines, compared with that of the corresponding intronless CDS. Thus, the long intron 1 of *GH* gene in Reeves’ turtle might correlate with downregulated gene expression.

## 1. Introduction

Reeves’ turtle (*Chinemys reevesii*, also known as *Mauremys reevesii*) belongs to the order Testudinata in the family Bataguridae and is found extensively in China, Japan, Korea, and other parts of Asia. In general, turtles are long-lived, slow-growing animals, and they are considered an important aquatic source of food in traditional Chinese medicine. Whether a relationship exists between turtle longevity and growth speed remains an open question. Additionally, the causal connection between slow growth and *growth hormone* (*GH*) gene alterations and expression patterns remains undefined. In previous studies on the microstructure of its pituitary gland, the ultrastructure of its adenohypophysis, and its blood biochemical indices [1,2], we found a higher nucleoplasmic ratio and fewer secretory granules in pituitary GH cells in Reeves’ turtle compared to other animal species. We argued that the turtle’s slow growth might correlate with this reduced number of secretory granules, which might be related to reduce *GH* expression and gene alterations.

Initial research on the *GH* gene cluster focused on mice and humans. Thereafter, *GH* genes from other species, including buffalo/cattle, sheep, pigs, and fish, were cloned and investigated. *GH* genes are primarily expressed in the anterior pituitary glands of all vertebrates and are involved in the regulation of nitrogen, lipid, carbohydrate, and mineral metabolism. In addition, GH is involved in many physiological processes, such as sexual maturation and immune function [3,4]. GH synthesis and secretion are regulated by hypothalamic neuropeptides, including growth hormone–releasing hormone (GH-RH) and somatotropin release-inhibiting factor (SRIF). Growth hormone and insulin-like growth factor-1 (IGF-1), GH’s actions, exert negative feedback on GH secretion. The GH/IGF-1 axis is a vital regulator of growth and ageing. In mice, attenuation of the GH/IGF-1 axis leads to increased lifespan [5]. However, variations in GH expression are also correlated with *GH* gene organization. Several studies have reported that selected fragments within the promoter and intron regions of the *GH* gene may regulate its transcription. Such regulatory sequences in the introns of the *GH* gene may be recognized by pituitary-specific transcription factor 1 (Pit-1), also known as growth hormone factor 1 (GHF-1) [6]. In a previous study investigating the transcriptional regulation of the gilthead sea bream (*Sparus aurata*), the results from transfection assays using the *growth hormone* (*saGH*) gene suggested that long introns within this gene may influence its expression *in vivo* [7]. In particular, regulatory elements contained in intron 1 of this gene may cooperate to regulate its cell type-specific expression at the transcriptional level [8,9,10]. Conserved sequences in intron 1 contain binding motifs for selected transcription factors. When these regions were deleted by homologous recombination in knockout mice, *GH* gene mRNA levels diminished [11]. Additionally, portions of the *GH* gene intron 1 sequence have been positively correlated with reporter gene expression and have been shown to stimulate transcription of the gene [12,13]. Conversely, a small fraction of the intron 1 sequence has been associated with reduced reporter gene transcription [14].

*C. reevesii* possesses fewer GH secretory granules than other animal species. Therefore, in the current study, we cloned the *GH* gene of this species and investigated specific similarities and differences in its sequence using four cell lines, including DF-1 chicken embryo fibroblasts, Chinese hamster ovary (CHO) cells, human embryonic kidney 293FT cells, and GH4C1 rat pituitary cells. In these cell lines, we compared the expression of a *GH* intron 1-containing coding sequence (CDS) with that of a corresponding intronless CDS. We found that long intron 1 in the *GH* gene can have both positive and negative effects on *GH* gene expression, and there is evidence to support the hypothesis that this intron may directly regulate *GH* expression.

## 2. Results

### 2.1. GH Cells in Reeves’ Turtle Pituitary Glands Contain Few Secretory Granules

**Figure 1:**
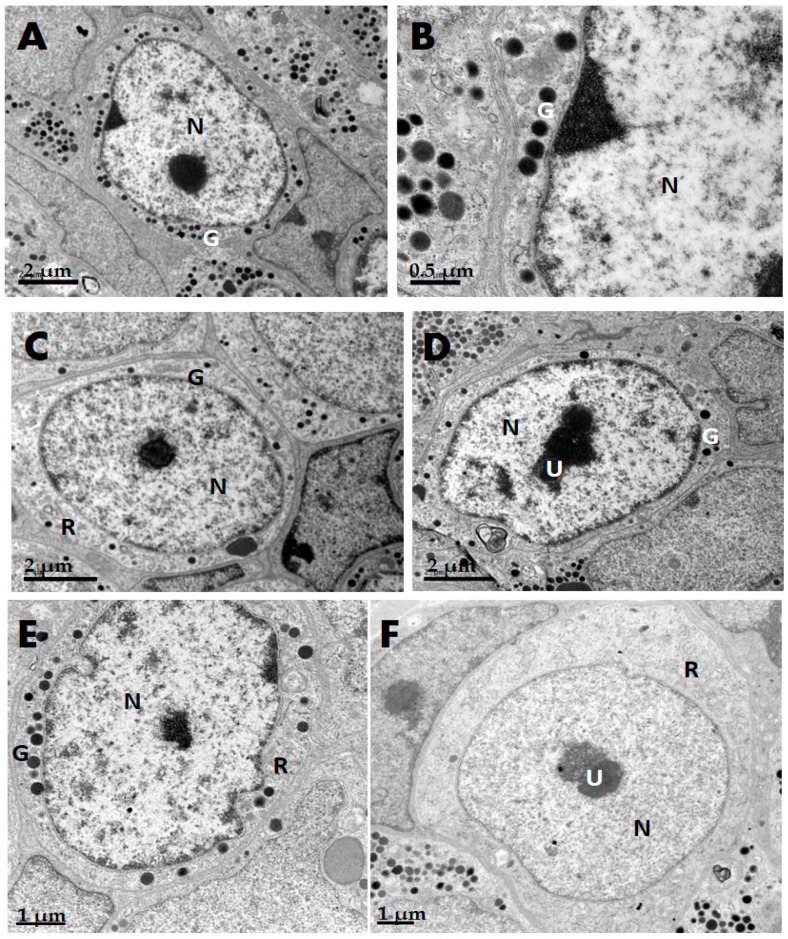
*Chinemys reevesii* pituitary GH cell ultrastructure in the winter. (**A**) A GH cell showing a few secretory granules and a big nucleus; (**B**) An enlarged section of (**A**) showing secretory granules in the cytoplasm; (**C**) appearance of the rough endoplasmic reticulum and ribosomes in GH cells; (**D**) nucleoli located in the center of a nucleus; (**E**) a GH cell showing depressed border of nucleus and scarce secretory granules; (**F**) a GH cell with no observable secretory granules. N, Nuclei; G, Secretory granules; R, Rough endoplasmic reticulum; U, Nucleoli.

During the growth season, GH cells collected from Reeves’ turtle pituitary glands were round in shape. Each nucleus appeared globular and contained one nucleolus. The nucleoplasmic ratio was high. A few small round secretory granules could be observed in the cytoplasm; they had blurred edges and uneven electron densities. In the winter, the GH cells had a few secretory granules and enormous nucleus (Figure 1A,B); some cells had no granules. Using Photoshop image-editing software, the figures were checked randomly and surveyed digitally. As a result, the nucleus and cell ratio of GH cells was 0.6726 ± 0.0018 (*n* = 6) and the secretory granule and cytoplasm ratio of GH cells was 0.0703 ± 0.0020 (*n* = 6) in Reeves’ turtles in the winter; it was 0.4941 ± 0.0099 (*n* = 6) and 0.2218 ± 0.0055 (*n* = 6), respectively, in the summer, as assessed by measuring the total area in each photograph. In the cytoplasm, the rough endoplasmic reticulum was observed to be randomly arranged in lamellae near the membrane, and there were several areas of dispersed rough endoplasmic reticulum in proximity to the nucleus. Each of these regions enlarged into a cisterna, on the edge of which were a few ribosomes (Figure 1C). Few mitochondria were present, and those that were present had a loosely organized structure. A fraction of mitochondria had a concentric circle appearance without a double membrane. The mitochondria also exhibited incomplete transverse ridges. The cell nuclei were orbicular-ovate, and the edges of the nuclear membranes were clear. The distribution of chromatin was uniform. There were one or two nucleoli in the center of each nucleus (Figure 1D). Some GH cells showed a depressed border of nucleus. Pitting was evident; in proximity to this, there were a few secretory granules. The observed secretory granules and pittings had approximately equal diameters (Figure 1E). Some of the turtle cells initially possessed no observable secretory granules (Figure 1F).The Reeves’ turtle pituitary cells have fewer growth hormone secretory granules in the winter than in the summer. However, secretory granules were usually present in the cells of other animals such as *Oreochromis nilotica*, *Silurus meridionalis*, SI-JI goose, and the beagle (dog) even during the winter (Figure S1).

### 2.2. Sequence and Characteristics of the Reeves’ Turtle GH Gene

**Figure 2:**
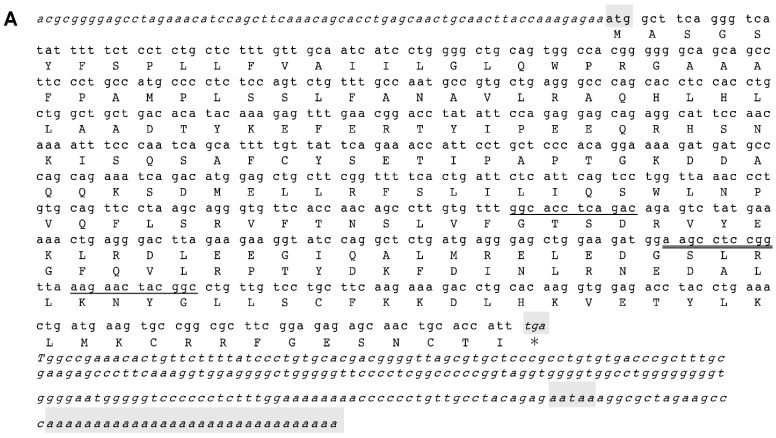

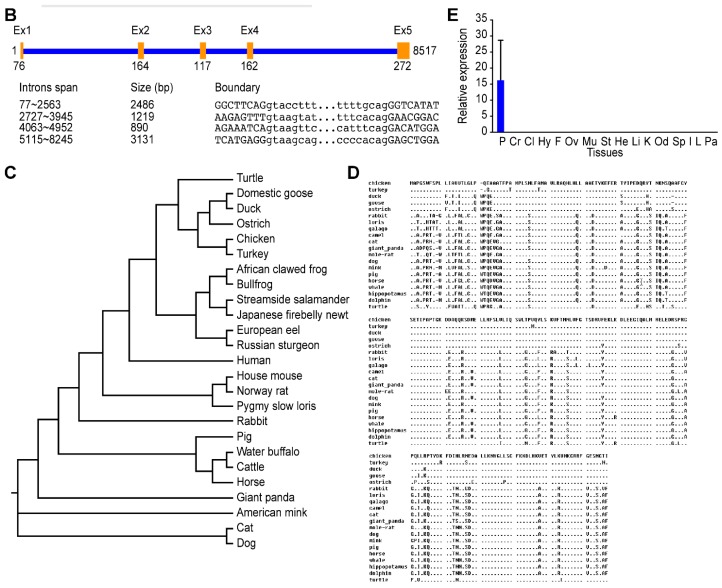
*Chinemys reevesii GH cDNA* sequence and gene characteristics. (**A**) The italic letters represent the 5′- and 3′-untranslated regions. The lowercase letters represent the coding sequence, with the corresponding protein sequence below. The shadowed letters represent ATG, TGA, tail signals, and the polyadenylation sequence aataaa in the 3′-region. Capital letters show amino acids for each codon upside, whereas * refers to stop codon. The primers GHF and GHR are single-underlined. The *TGSP1* and *TGSP2* 5′-RACE and 3′-RACE primers are double-underlined; (**B**) Structural characteristics of *C. reevesii GH* gene. Capital letters are exon sequences; lower case letters are intron sequences; (**C**) Phylogenetic tree representing *GH cDNA* from various animal species; (**D**) Comparison of GH amino acid sequence homology in different species; (**E**) *GH* gene expression in various tissues from *C. reevesii*. P, pituitary; Cr, cerebrum; Cl, cerebellum; Hy, hypothalamus; F, fat; Ov, ovary; Mu, muscle; St, stomach; He, heart; Li, liver; K, kidney; Od, oviduct; Sp, spleen; I, small intestine; L, lung; Pa, pancreas.

In this study, full-length *GH* cDNA (NCBI accession number: EF424785) of *C. reevesii* was cloned for the first time. Furthermore, this was the first successful cloning of a *GH* gene from any reptilian species. The Reeves’ turtle *GH* gene was found to have a length of 791 bp, containing a 66-bp 5′-untranslated region (UTR), a 654-bp open reading frame, and a 71-bp 3′-UTR. The gene encodes a 217-amino-acid peptide (NCBI accession number: ABO09819.1), comprising a 26-amino-acid signal peptide and a 191-amino-acid mature peptide (Figure 2A). The GH protein has a molecular weight of approximately 24,856.68 Da and an isoelectric point of 7.78. In addition, the full-length *GH* gene, which is 8517 bp in length, was isolated (NCBI accession number: EU647240). The full-length gene included five exons with lengths of 76, 164, 117, 162, and 272 bp; and four introns with lengths of 2486, 1219, 890, and 3131 bp (Figure 2B). Typical intron–exon junction structures with donor (GT) and acceptor (AG) dinucleotide sequences were conserved in the turtle *GH* gene. The total length of all introns was 7726 bp; this is one of the longest introns found in animal *GH* genes to date.

The proportions of GC and AT in the *GH* cDNA sequence of *C. reevesii* were 50.46% and 49.54%, respectively. In mammals and birds, *GH* cDNA had a higher GC content than AT content: in pigs, the gene’s GC content was found to be 62.06%; in buffalo/cattle, it was 59.94%; in goats, it was 59.63%; in mice, it was 57.76%; and in humans, it was 56.12%. In contrast, *GH* cDNA in amphibians and fish had a higher AT content than GC content: in bullfrogs, the gene’s GC content was found to be 44.29%; in rainbow trout, it was 40.55%; and in salmon, it was 39.13%. The coding region of the Reeves’ turtle *GH* gene contained 50.46% GC, which was lower than that in mammals and birds and higher than that in amphibians and fish. This intermediate proportion corresponds to patterns of biological evolution, in which reptiles evolved sometime between birds and amphibians. The coding sequence of the Reeves’ turtle *GH* gene shared 85.0%, 83.4%, 83.4%, 83.3%, and 82.5% nucleotide identity with the *GH* genes from the ostrich, duck, chicken, goose, and turkey; and 86.2%, 84.7%, 83.3%, 84.7%, and 81.0% amino acid sequence identity, respectively. In addition, the Reeves’ turtle *GH* cDNA shared similarities of 74.8%, 72.5%, 70.2%, 65.9%, and 64.1% with horse, mouse, bullfrog, sturgeon, and human *GH* cDNA, respectively. These data indicate that the *GH* gene was conserved during evolution. The greatest similarity in *GH* cDNA was found between Reeves’ turtle and birds (Figure 2C). The Reeves’ turtle GH amino acid sequence was also highly similar to certain other animals, sharing 99.0% and 92.6% similarity with *Chelydra* and the green sea turtle, respectively. The GH amino acid sequence had a comparable similarity (Figure 2D). Given the phylogenetic position of reptiles between mammals and amphibians, characterizing the molecular structure of the *GH* gene in *C. reevesii* should be useful in interpreting evolutionary trends.

As determined by qRT-PCR, *GH* mRNA expression was detected only in the pituitary gland and was undetectable in the cerebrum, cerebellum, hypothalamus, lung, heart, liver, pancreas, spleen, stomach, small intestine, kidney, ovary, oviduct, muscle, and fat (Figure 2E). Therefore, *GH* was uniquely expressed in the pituitary gland.

### 2.3. Effect of Intron 1 on Reeves’ Turtle GH CDS Expression in Four Cell Lines

**Figure 3:**
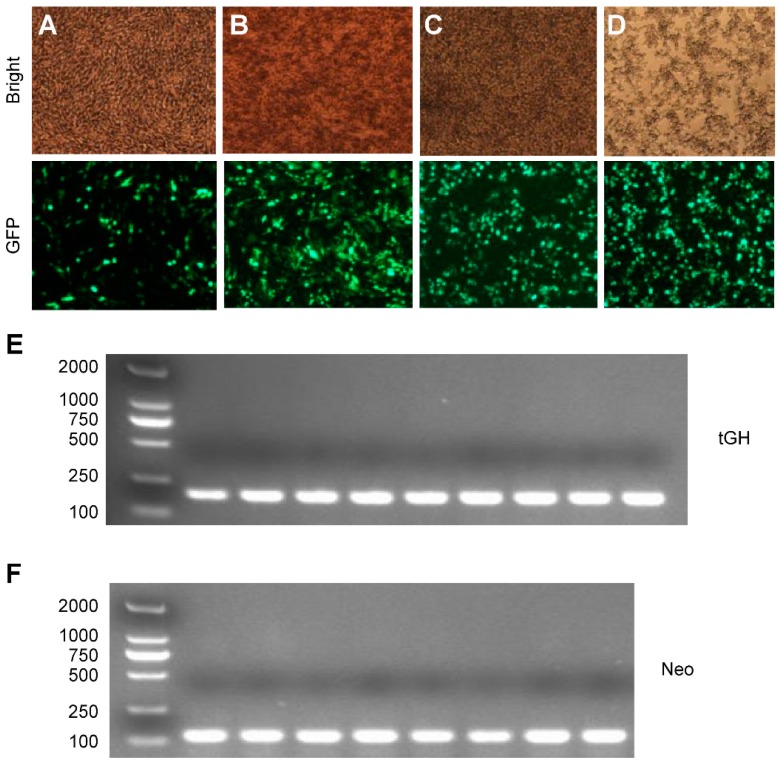
Cells at 48 h post-transfection with pcDNA3.1-EGFP and the RT-PCR product formed following the induction of Reeves’ turtle GH gene expression. (**A**) DF-1 cell line; (**B**) CHO cell line; (**C**) 293FT cell line; (**D**) GH4C1 cell line. Each cell line was transfected with 2 μL of Lipofectamine and a total of 1200 ng of DNA (40×); (**E**) Electrophoresis map showing the PCR products generated from the transfected cell lines. The lanes showing pcDNA3.1tGH-6H and pcDNA3.1tGH-in-6H were chosen at random from the four cell lines; (**F**) Electrophoresis map of the *neo* gene PCR product. All of the vectors contained the *neo* gene, which served as an internal transfection control.

The effects of intron 1 on Reeves’ turtle *GH* gene expression were examined in four cell lines: DF-1, CHO, 293FT, and GH4C1. To assess these effects, genomic DNA and mRNA were isolated from the blood and pituitary glands of turtles, and the *GH* CDS was then PCR-amplified using cDNA as a template. A sequence containing both the CDS and the first intron was amplified with overlapping PCR using both genomic DNA and cDNA as templates. DNA sequencing was used for validation. Vectors were constructed by ligating the PCR fragment into a plasmid and tested by restriction enzyme digestion. Diagrammatic representation of the two vectors was shown in Figure S2. The electrophoresis maps showed that the vectors were correct (Figure S2).Real-time PCR was used to detect *GH* mRNA expression following transfection of the vector into the four cell lines. As shown in Figure 3A–D, pcDNA3.1-EGFP was transfected into the four cell lines to optimize the transfection conditions. Because all of the vectors contained the *neo* gene, the *neo* gene PCR product served as an internal transfection control. Furthermore, β-*actin* mRNA was used as an internal expression control. All primers and cDNA samples were evaluated using gel electrophoresis; the electrophoresis maps are shown in Figure 3E,F.

**Figure 4:**
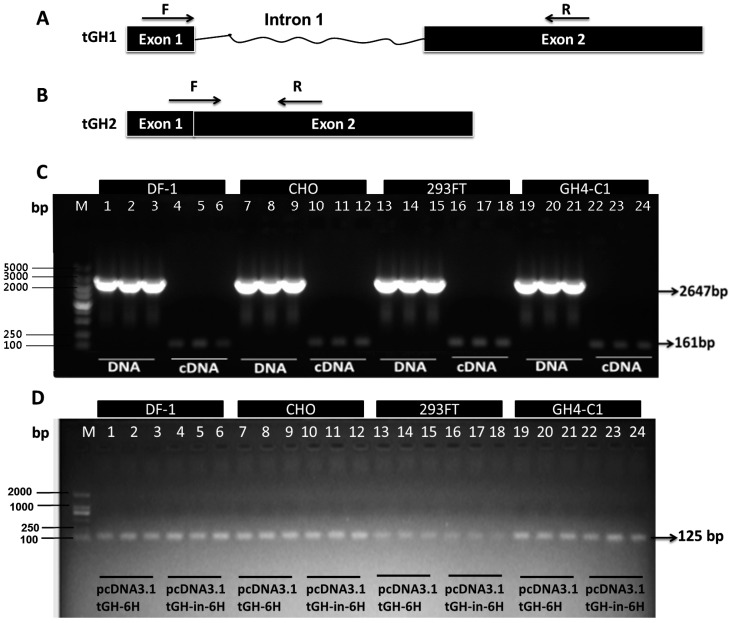
PCR product confirms the correct splicing of *tGH* intron 1 in DF-1, CHO, 293FT, and GH4-C1 cell lines at 48 h post-transfection with pcDNA3.1tGH-6H or pcDNA3.1tGH-in-6H. (**A**) Schematic diagram showing the position of tGH1 primes in *tGH* gene, which were depicted by arrows. Forward primer was located on exon 1, and reverse primer was located on exon 2; (**B**) Schematic diagram showing the position of tGH2 primes in *tGH* gene, which are depicted by arrows. Forward primer was located at the junction of exon 1 and exon 2, and reverse primer was located on exon 2; (**C**) Electrophoresis map showing the PCR product generated from the four cell lines post-transfection with pcDNA3.1tGH-in-6H. tGH1-F and tGH1-R were used as the primers, which were depicted in (**A**). DNA or cDNA from the four cell lines was used as template, marked by the white bold characters; (**D**) Electrophoresis map showing PCR products generated from the four cell lines post-transfection with pcDNA3.1-tGH-6H or pcDNA3.1-tGH-in-6H. tGH2-F and tGH2-R were used as the primers, depicted in Figure 4B. The temples were cell cDNA from the four cell lines transfected with pcDNA3.1-tGH-6H or pcDNA3.1-tGH-in-6H, which are marked by white or black bold characters. The lanes show the cell samples chosen at random from the four cell lines in (**C**,**D**).

To prove the efficient splicing of the *tGH* intron 1 in DF-1, CHO, 293FT, and GH4-C1 cell lines at 48 h post-transfection with pcDNA3.1tGH-in-6H, it was detected whether the intron 1 sequence was retained in the transcriptional products. DNA and RNA were extracted from the transfected cells and exogenous sequences were identified by PCR amplifying, using DNA or cDNA as the template, and designed tGH1 primers as shown in Figure 4A. As the PCR amplification results shown in Figure 4C, the length of product was a 2647-bp-fragment based on DNA as a template, while the length of product was 161 bp using cDNA as a template. It showed that the transcriptional products did not retain the intron 1 sequence in the four cell lines transfected with pcDNA3.1tGH-in-6H. To further analyze the accurate splicing of the intron 1 in the *tGH*, we investigated part of its mRNA sequence in the four cell lines transfected with pcDNA3.1tGH-6H or pcDNA3.1tGH-in-6H plasmids (Figure 4D). Using cell cDNA as the temple, tGH2 primers shown in Figure 4B were designed to detect the first intron spliced correctly. We cloned a 125-bp fragment of the *tGH* gene in the cell group transfected with pcDNA3.1-tGH-6H or pcDNA3.1-tGH-in-6H plasmids (Figure 4D). These results show that the *tGH* intron 1 has been spliced correctly and efficiently in the four cell lines.

Expression of the plasmids pcDNA3.1tGH-6H and pcDNA3.1tGH-in-6H was compared across all four cell lines. The tGH-in-6H fragment contained all of intron 1 and the CDS from the Reeves’ turtle *GH* gene, while the tGH-6H fragment contained only the CDS.

**Figure 5:**
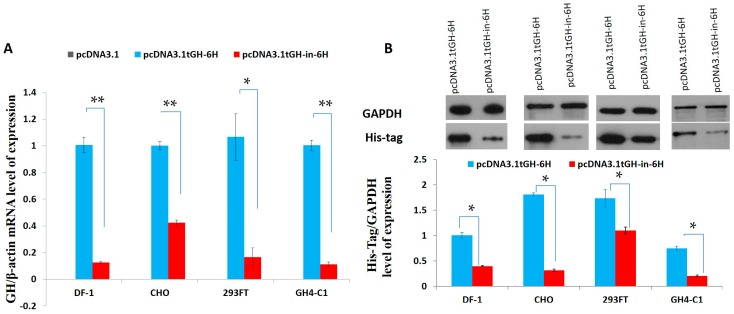
*GH* gene mRNA and protein expression following the transfection of four cell lines. (**A**) The mRNA expression level with qRT-PCR test after the four cell lines were treated with vectors for 48 h. The expression of turtle GH mRNA transfection with pcDNA3.1tGH-in-6H was only 13%, 42%, 17%, and 11% of the pcDNA3.1tGH-6H expression level in DF-1, CHO, 293FT, and GH4-C1, respectively (*p* < 0.05); (**B**) The protein expression level with Western blot test after the four cell lines were treated with vectors for 48 h. The expression of turtle GH transfection with pcDNA3.1tGH-in-6H were only 39%, 18%, 63%, and 27% of the pcDNA3.1tGH-6H expression level in DF-1, CHO, 293FT, and GH4-C1, respectively. Data were processed using the statistical software package SAS 9.1.3 (SAS Institute Inc., Cary, NC, USA) and expressed as the mean ± SEM. Variance analysis was operated using GLM procedure, based on at least three replicates for each treatment. * *p* < 0.05 compared between the two indicated constructs; ** *p* < 0.01 compared between the two indicated constructs.

The expression of tGH-in-6H were only 13%, 42%, 17%, and 11% of the pcDNA3.1tGH-6H expression level in DF-1, CHO, 293FT, and GH4C1, respectively (*p* < 0.05) (Figure 5A). The mean expression level of the His-tagged protein was recorded by Western blot assays. The expression of tGH-in-6H were only 39%, 18%, 63%, and 27% of the pcDNA3.1tGH-6H expression level in the four cell lines (Figure 5B).

## 3. Discussion

Turtles are cold-blooded animals that hibernate from November through March or April of the next year. *C. reevesii* grows slowly in nature. Its growth rate is the fastest between 3 and 4 years of age. It reaches sexual maturity at 5 years, after which it grows more slowly because most of its nutrient intake is used for egg cell development in females. GH cells in the pituitary gland have similar characteristics across different species, such as *Oreochromis niloticus* [15], SI-JI geese [16], beagle dogs, and Rhesus monkeys [17] (Table 1). For example, GH cells in beagles are round, large, and contain many round secretory granules. In the present Reeves’ turtle samples, the electron density of the cytoplasm was high. Additionally, a few areas of rough endoplasmic reticulum were observed, as well as mitochondria, free nuclei, lysosomes, and other features. In the samples, the nuclei were located in the centers of the cells and had low electron density. The cells were enriched in euchromatin but had little heterochromatin. There were few secretory granules, and some cells had no observable secretory granules in the winter. The nuclei appeared to be large. In mature Reeves’ turtle GH cells, the number of secretory granules is representative of various cell functions, as these cells are involved in regulating development. Therefore, the number of GH cells, the number of secretory granules within these cells, and the degree of secretion all play vital roles in animal growth. To date, the relationship between various characteristics of GH cells in the turtle pituitary gland and cell synthesis and secretion, which might be driven by *GH* gene expression, has not been reported.

GH was previously thought to be primarily synthesized and secreted by pituitary somatotropes, but it is now well established that the *GH* gene can be expressed in many extrapituitary tissues, such as in the head kidney of the gilthead sea bream and in the brain, gill, heart, kidney, liver, pyloric caeca, and ovary of the rainbow trout [20]. Furthermore, GH has been detected in extrapituitary tissues in mammals and birds [21,22,23,24]. Therefore, GH is derived not only from the pituitary but also from extrapituitary tissues in a paracrine manner or through secretion. However, in the current study, no *GH* gene expression was detected in 15 non-pituitary tissues from *C. reevesii*. Thus, Reeves’ turtle GH is uniquely expressed in the pituitary.

The molecular mechanism underlying Reeves’ turtle GH expression is potentially very interesting. In the *C. reevesii GH* gene, the length of its first intron was found to be 2486 bp, which is significantly greater than that of other vertebrates (Table S1). Intron length can affect gene expression. Normally, in highly expressed genes, introns are substantially shorter than those in genes expressed at low levels [25]. In the referenced study, the regulatory region of the *AChE* gene, corresponding to a conserved sequence in the gene’s first intron, was deleted in mice by homologous recombination. The knockout mice were virtually devoid of AChE activity and *AChE* mRNA in skeletal muscle, yet AChE activity in the brain and spinal cord, which innervated the skeletal muscle, were unaltered. Based on these findings, it would be worthwhile to translate the current study’s findings into transgenic animals to further evaluate the function of the first intron in the Reeves’ turtle *GH* gene. Because strong viral control elements (e.g., the CMV promoter and the SV40 poly A) were chosen, the *GH* gene CDS could be expressed in both pituitary and non-pituitary cell lines [26,27]. Instead of reporter gene assays, the CDS of the *GH* gene was evaluated. The original position of the first intron was retained in the vectors. RNA from cells transfected with these vectors was extracted and then used PCR to show that intron 1 could regulate *tGH* expression [28,29].Therefore, there is a clear basis for the hypothesis that slow growth in turtles is correlated with low *GH* expression and that low *GH* expression is associated with the length of intron 1.

Evidence showing scarce growth hormone secretory granules in the pituitary glands of turtles and reduced expression of the *GH* was obtained in both our previous study [1] and the present study. Firstly, intron length is potentially associated with gene splicing. The process of *GH* expression might slow down when the first intron is present *in vivo*. As genes evolve, their introns tend to shorten [25]. In our study, intron 1 from the Reeves’ turtle *GH* gene appeared to affect *GH* transcription efficiency and inhibit *GH* expression; these effects may have been related to the length of intron 1. Following the transfection of the intron 1 sequences into various cell lines, a repressive effect on *GH* expression was observed, indicating that intron 1 is involved in gene regulation. Examination of the first intron sequence of the Reeves’ turtle *GH* gene revealed several GC response element (GRE) half-sites (Figure S3). The GRE motif (GCTACAnnnTGTTCT) [30] is a canonical glucocorticoid response element.Half of the identified GREs also alter reporter gene activity [31,32]. Therefore, additional studies are required to identify the mechanism by which the first intron of the *GH* gene regulates expression. Motifs within the first introns of several genes play major roles in regulating gene expression. Intron 1 in the *AChE* gene contains an N-box motif, which is an intronic enhancer [12]. Elements in the untranslated first exon or first intron of the *CPT-I*β gene might influence the responsiveness of this protein to hypertrophic stimulus [13]. *ABCC6* gene expression is regulated by the activation of a primate-specific sequence located in the first intron of the gene via the CCAAT/enhancer binding protein [10]. All of these studies demonstrate that special sequences contained within first introns can bind various factors and increase gene expression. In Reeves’ turtle, the first intron of the *GH* gene had a negative effect on gene expression in four cell lines. Studies on the function of the first intron in the *Sparus aurata* growth hormone (*saGH*) gene have indicated that the long and forward-oriented intron represses the transcriptional activity of reporter genes in both mammalian and fish cell lines [33]. Therefore, we speculate that the first intron of the Reeves’ turtle *GH* gene should have a repressive effect on gene expression.

In several animal models, reduced levels or activities of *GH* and *IGF-1* have been related to significant increases in both average and maximal lifespan [34]. GH-resistant and GH-deficient mutant mice have substantially increased lifespans [35]. Increased lifespans are also observed in animals with isolated GH deficiency, such as Laron dwarfs [36]. Ames dwarf mice live approximately 50% longer than their normal siblings [37]. In humans, reduced life expectancy has been observed in patients with either severe GH deficiency or GH excess, although these cases could be explained by increased risks of cardiovascular disease, diabetes, or cancer rather than because of an acceleration of the ageing process [38]. There is consistent evidence for the pro-longevity effects and ability of these interventions to prevent or delay multiple age-related diseases and improve health span. A reduction in IGF-1 levels or *IGF-1* action can extend lifespan in animal models [39]. During aging, some hypermethylated sequences are found within gene promoters, leading to alterations in gene expression that could impact on the regenerative capacity of a cell [40]. In the Kaplan–Meier analysis, females with IGF-1 levels below the median had significantly longer survival than that above the median. Lower IGF-1 levels predicted longer survival in both males and females with a history of cancer [41]. *C. reevesii* is a slowly growing animal. Generally, turtles possess fewer secretory granules in GH cells, which is a probable cause of this slow growth. In addition, these low levels of IGF-1 in turtles [2] may slow growth in favor of prolonged longevity. However, the platelet-derived growth factor-D over-expression significantly promoted tumor growth and the invasion occurred both *in vitro* and *in vivo* [42]. Recently, Ghrelin stimulating growth hormone secretion and regulating appetite is discovered in fish and mammals. Its expression was significantly upregulated from fasting and downregulated after refeeding [43]. Moreover, the expression of miR-125b in mitochondrial fractions showed a significant downregulation after administration of recombinant human growth hormone [44].

To date, no *in vitro* or *in vivo* data have been presented on the function of intron 1 in Reeves’ turtles or any other species of turtle. However, previous research has shown that *C. reevesii* has fewer GH secretory granules than other animals. In this study, we cloned and investigated the Reeves’ turtle *GH* gene and found that its first intron was much longer than those of other species. The mRNA expression of a first intron-containing *C. reevesii GH* gene CDS was only 13%, 42%, 17%, and 11% of an intronless *GH* CDS in the DF-1, CHO, 293FT, and GH4-C1 cell lines, respectively. Additionally, transfection with pcDNA3.1tGH-in-6H showed 39%, 18%, 63%, and 27% *tGH* protein expression level of pcDNA3.1tGH-6H in various cells lines. The long intron 1 of *growth hormone* gene from *C. reevesii* correlates with repression of GH expression. The negative effects of the intron might downregulate *GH* expression. Therefore, *C. reevesii* is a suitable animal model for research into molecular mechanisms of lifespan.

## 4. Experimental Section

### 4.1. Sample Preparation

All turtles (ranging from 410 to 655 g in body mass) used in this study were purchased from the Guangzhou Fangcun animal market. The turtles were housed in 20 L aquarium tanks containing filtered water and maintained under normal husbandry conditions.An ambient temperature of 12–13 °C was maintained for one week in winter. Three turtles were taken to evaluate the ultrastructures of GH cells in the pituitary gland. Three turtles were used for cloning, sequencing, and real-time reverse transcription polymerase chain reaction (RT-PCR) analysis of the *GH* gene. Finally, three additional turtles were used to study *GH* gene mRNA and protein expression via transfection of the above-described cell lines. The turtles were in good health prior to sample collection, and they were sacrificed with anesthesia as necessary to ameliorate suffering in accordance with our institution’s guidelines for experimental animal care.

### 4.2. Ultrastructure of Pituitary Gland GH Cells in C. reevesii

Pituitary glands were collected from the turtles, fixed in 10% Bouin solution immediately after excision, and embedded in paraffin. Next, the samples were sliced into 4-μm-thick sections, deparaffinized and rehydrated, and hematoxylin & eosin (HE) staining was used to visualize endogenous peroxidase. The sections were subsequently mounted on slides using neutral gum and visualized using a microscope. Additional pituitary gland samples were fixed with 4% glutaraldehyde for 24 h at 4 °C, washed with phosphate-buffered saline (PBS) (0.2 mol/L, pH 7.4) for 2 h, fixed with 1% osmic acid for 2 h, and then washed six times with PBS for 10 min per wash. Following this, the samples were dehydrated with ethanol and cleaned with epoxypropane. They were then embedded in EPON 812 overnight at room temperature. Ultrathin sections (1 μm) were sliced to examine localization patterns via electron microscopy. These sections were obtained using a Leica UCT ultramicrotome and stained with 1% toluidine blue. Additional ultrathin sections (40–60 nm) were obtained with the Leica UCT and then stained with uranyl acetate/lead citrate. These sections were subsequently visualized using a FEI Tecnai T12transmission electron microscope (Hillsboro, OR, USA). More than 100 cells were examined and showed the features from turtle pituitary glands.

### 4.3. Tissue Collection and Isolation of DNA and RNA

Genomic DNA samples were isolated from turtle blood using a phenolic extraction protocol. These samples were used to clone the *GH* gene. To determine tissue-specific gene expression patterns, the following 16 tissue types were removed from the turtles: pituitary gland, cerebrum, cerebellum, hypothalamus, fat, ovary, muscle, stomach, heart, liver, kidney, oviduct, spleen, small intestine, lung, and pancreas. Following collection, the tissues were immediately frozen in liquid nitrogen and stored at −80 °C before RNA extraction. Total RNA was isolated from 0.2 g of skeletal muscle tissue using TRIzol^®^ (Invitrogen Life Technologies, Carlsbad, CA, USA) and an RNeasyMinElute Cleanup Kit (QIAGEN, Beijing, China) according to the manufacturer’s instructions. All RNA samples were quantified by a NanoDrop 2000c (Thermo, Waltham, MA, USA). RNA purity and yield were determined based on optical density at 260 and 280 nm. RNA integrity was assessed by electrophoresis on a 1.2% denaturing formaldehyde gel.

### 4.4. Cloning, Homology Analysis, and Tissue-Specific Expression Patterns of the Reeves’ Turtle GH Gene

*GH* gene sequences from mammals, birds, amphibians, and fish were downloaded from the National Center for Biotechnology Information (NCBI). *GH* gene sequences from 16 species, including humans, mice, bullfrogs, eels, and chickens, were used to identify conserved sequences with ClustalX. Primers were designed against sequences encoding GH factor and GH receptor (GHF and GHR) in a conserved area for partial complementary DNA (cDNA) cloning of the Reeves’ turtle *GH* gene. Then, according to the partial cDNA sequence, primers (TGSP1 and TGSPR2) were designed for 5′- and 3′-rapid amplification of cDNA ends (RACE). Primers GP2 and GP3 were designed for RT-PCR. GP2 was designed based on full-length Reeves’ turtle *GH cDNA* (GenBank accession number: EF424785) and spanned introns 3 and 4 to control for DNA contamination. GP3 was designed based on chicken β-*actin* (GenBank accession number: L08165.1) and was used as an internal control. Primers GIP1, GIP2, GIP3, and GIP4 were designed based on full-length *GH cDNA* and were used to clone introns 1, 2, 3, and 4, respectively (Table S2).

### 4.5. RT-PCR, RACE, and DNA Sequencing

Reverse transcription was performed using a reaction mixture that included 2 μg of RNA, 5 μL of 5× reverse transcription buffer, 2 μL of 2.5 mmol/L deoxynucleotide triphosphates (dNTPs), 2 μL of 100 mmol/L dithiothreitol, 4 μL of 8 U/μL RNasin, 2 μL of 50 pmol/L anchor primer, and sufficient diethyl pyrocarbonate-treated water for a total volume of 24 μL. The mixture was incubated at 64 °C for 5 min, 37 °C for 60 min (1 μL of 200 U/μL M-MLV enzyme was added after 10 min), and 95 °C for 5 min; it was then cooled on ice. First-strand DNA was used for both RT-PCR and quantitative RT-PCR (qRT-PCR) analysis. cDNA from the pituitary gland was used as a PCR template. The Reeves’ turtle *GH* gene was amplified using the primer GP1. PCR was performed in a total volume of 25 μL, comprising 0.5 μL of first-strand cDNA, 2.5 μL of 10× Ex Taq PCR buffer, 4 μL of 2.5 mmol/L dNTPs, 0.5 μL of a 20 pmol/L mixture of GP1F and GP1R, 0.25 μL of Ex Taq enzyme, and 17.25 μL of sterile deionized water. The PCR conditions were as follows: 3 min at 94 °C; 35 cycles of 30 s at 94 °C, 30 s at 46 °C, and 45 s at 72 °C; and a final extension of 5 min at 72 °C in a Mastercycler Gradient (Eppendorf Limited, Hamburg, Germany). The PCR products were purified using an Agarose Gel DNA Purification Kit 2.1 and then inserted into a PMD18-T vector (Takara Bio Inc., Dalian, China). Positive clones of the expected sizes were sequenced using the GP1 primer. The RACE Core Set (Takara Bio Inc.) was used to produce 5′- and 3′-RACE cDNA with the gene-specific primers (Table S2) TGSP1 and TGSP2, respectively. Reverse transcription reactions were performed with 3–5 μg of total RNA isolated from the pituitary gland. The RACE products were purified, cloned into PMD-18T vectors (Takara Bio Inc.) and sequenced.

### 4.6. Homology Analysis

Data corresponding to GH proteins and coding sequences from 20 species were downloaded from the NCBI website. The resulting sequences were confirmed using the BLAST program on the NCBI server [45]. SeqMan software (DNASTAR, Madison, WI, USA) was used to splice the cDNA and terminal sequences obtained from RACE. The amino acid sequence of the *GH* gene was deduced using the ExPaSy translate tool, and its physicochemical properties were predicted using the ExPaSyProtParam tool [46]. The presence of signal peptides in the GH protein was analyzed using the SignalP 3.0 server [47]. TMHMM2.0 software was used to predict transmembrane domains [48]. A phylogenetic tree was constructed using the MEGA software package, version 4.0 [49].

### 4.7. Plasmid Construction

All primers in this study were designed using Gene Tool, and restriction sites were identified using NEBcutter (New England BioLabs Inc., Beijing, China). Genomic DNA was extracted from turtle blood. RNA was extracted from turtle pituitary gland tissue. tGH fragments contained only the turtle *GH* CDS, whereas tGH-in fragments consisted of a short *GH* CDS and the first intron of the turtle *GH* gene (Figure S4). These fragments included 10 bp of the 3′ end of the first exon, the first intron and the rest of the CDS. Sequences were amplified using primers that contained restriction enzyme sequences (Table S3). DNA fragment (Table S4) ends were filled in with sequences encoding six repeating histidines; this His-tag was used to evaluate protein expression.

*GH* CDS plasmids (named pcDNA3.1tGH-6H) were constructed by replacing the sequence between the Hind III (+912) and EcoRI (+953) restriction sites in the pcDNA3.1(+) vector (Invitrogen) with the *GH* CDS and *GH*-in sequences generated by PCR. The pcDNA3.1tGH-in-6H plasmid included a sequence insertion between the NheI (+896) and EcoRI (+953) sites. Both constructs contained the *GH* CDS, whereas only the tGH-in construct contained intron 1. Intron 1 was located between exon 1 and exon 2 in the reconstructed vectors.

### 4.8. Cell Culture and Transfection

DF-1 chicken embryo fibroblasts and human embryonic kidney 293FT cells were grown in Dulbecco’s modified Eagle’s medium supplemented with 10% fetal bovine serum (FBS). GH4-C1 rat pituitary cells and CHO cells were grown in RPMI-1640 medium supplemented with 10% FBS. All cell lines were grown in a humidified atmosphere of 5% CO_2_ at 37 °C. For transfection assays, each of the four cell lines was individually transfected with each of six reconstructed vectors.

Each cell line was also transfected with pcDNA3.1(+). The cells were cultured in 60-mm plates (Corning Costar Corporation, Cambridge, MA, USA) until they reached 80%–90% confluence. One day before transfection, the cells were trypsinized and seeded into 12-well plates (Corning Costar Corporation, Cambridge, MA, USA) in the appropriate media without antibiotics. After a 24-h attachment period, the cells were transfected with uncut pcDNA3.1GH-6H, pcDNA3.1GH-in-6H, or pcDNA3.1(+) using Lipofectamine 2000 (Invitrogen) according to the manufacturer’s recommendations. Each cell line was transfected using 2 μL of Lipofectamine and 1200 ng of DNA. The amount of plasmid DNA and the ratio of plasmid DNA to Lipofectamine 2000 for these transfections were determined in preliminary experiments. Following transfection, the cultures were incubated for 6 h, the transfection medium was replaced with fresh medium containing all necessary supplements, and the cells were returned to the incubator. The cells were incubated for a total of 48 h post-transfection and then collected for analysis.

### 4.9. Real-Time Quantitative PCR Analysis

To evaluate tissue-specific *GH* gene expression patterns in Reeves’ turtle, qRT-PCR was performed using an Mx3005P Stratagene sequence detection system with SYBR Green PCR Master Mix (TOYOBO, Shanghai, China). First-strand cDNA from tissue samples collected from six selected turtles was used as a template. β-*actin*, a housekeeping gene, was used as an internal control. The GP2 and GP3 primers were used to amplify turtle *GH* and β-*actin*, respectively. Total RNAwas extracted from different transgenic cells using an HP Total RNA Kit (Omega, Stamford, CT, USA). RNA integrity and concentration were respectively assessed using denaturing gel electrophoresis and a NanoDrop 2000c (Thermo, Waltham, MA, USA). Total RNA from each sample was reverse-transcribed using a PrimeScript™ RT Reagent Kit (Perfect Real Time) (Takara). The following primer pairs were used: tGH-F and tGH-R for tGH, DF-1-F, and DF-1-R for β-*actin* in DF-1 cells; CHO-1-F and CHO-1-R for β-*actin* in CHO and GH4-C1 cells; and 293FT-F and 293FT-R for β-*actin* in 293FT cells. Neo-F and neo-R were used as an internal transfection control. tGH1-F, tGH1-R,tGH2-F, and tGH2-R were used for detecting the accurate splicing (Table S5).

Amplification was performed using a reaction volume of 20 μL with a mixture comprising 1 μL of cDNA, 0.2 μL of each primer, 10 μL of SYBR Green Real-time PCR Master Mix and 8.6 μL of ultrapure RNase-free water. The PCR was performed with the following parameters: 95 °C for 3 min; 40 cycles of 95 °C for 30 s, 63 °C for 30 s, and 72 °C for 45 s; 95 °C for 15 s; 60 °C for 20 s; and 95 °C for 15 s. The *GH* and β-*actin* genes were separately evaluated, and the reactions were repeated three times for each sample. To confirm product specificity, dissociation curve analysis was conducted. Relative *GH* gene expression was measured using the 2^−Δ*C*q^ method, where Δ*C*q indicated difference between the average cross threshold (*C*q) of the target gene and the average *C*q of β-*actin.* The results were expressed as the mean ± standard error.

### 4.10. Immunoblotting and Immunofluorescence

Immunoblotting was performed using standard procedures and antibodies against the included His-tag (Santa Cruz Biotechnology, Santa Cruz, CA, USA) and GAPDH (Bioworld, St. Louis Park, MN, USA).

### 4.11. Statistical Analysis

All data were analyzed using Student’s *t*-test. The data are presented as the mean ± SD. A probability *p*-value of <0.05 was considered statistically significant. To validate our results, all experiments were repeated at least two times.

### 4.12. Ethics Standards

Animal experiments were handled in strict accordance with guidelines approved by the Animal Care Committee of South China Agricultural University (Guangzhou, China) with approval number SCAU#0011, 3 August 2010.

## 5. Conclusions

There have been no data presented on the function of intron 1 in turtles of this or any other species *in vivo* or *in vitro* before. Our work showed that *C. reevesii* has fewer GH secretory granules than other animals. The turtle’s *GH* gene was cloned and it was found that its intron 1, in particular, was much longer than in other species. The coding sequence (CDS) of the turtle’s *GH* gene, with and without the inclusion of intron 1, was transfected into four cell lines, including DF-1 chicken embryo fibroblasts, Chinese hamster ovary (CHO) cells, human embryonic kidney 293FT cells, and GH4C1 rat pituitary cells; in these cell lines, the intron-containing CDS had 13%, 42%, 17%, and 11%, respectively, of the *tGH* mRNA expression level of the corresponding intronless CDS. Additionally, transfection with pcDNA3.1tGH-in-6H showed 39%, 18%, 63%, and 27% *tGH* protein expression level of pcDNA3.1tGH-6H in various cells lines. Intron 1 affected *GH* gene activity *in vitro*, which suggests that it might be involved in attenuation of the GH/IGF-1 axis. We concluded that the negatively regulated GH expression might correlate with the long sequence intron of the Reeves’ turtle *GH* gene.

## Figures and Tables

**Table 1:** Comparison of GH cell ultrastructure among different vertebrates.

Species	Cell Shape	Cell Diameter (μm)	Nucleus Shape	Nucleus Diameter (μm)	Secretory Granule, Number, Shape, Size (nm)	Organelle Morphology
Turtle	Round	8–10	Round	5–7	Few; rare in winter; diameter 250–300 nm	Mitochondria round or oval; a few Golgi apparatuses
*Oreochromis niloticus* [15]	Cuboidal or round	8–12	Round	4–5	Concentrated in winter, occupation in whole cytoplasm in early spring; diameter 230–350 nm	Mitochondria oval; many Golgi apparatuses; rough endoplasmic reticulum arranged in small fragments
Triangle bream [18]	Orbicular-ovate	11–13	Round	4–6	Many; diameter 220–440 nm	
*Silurus meridionalis* [19]	Irregular		Irregular		concentrated; 150–300 nm	Rough endoplasmic reticulum oval; many Golgi apparatuses
SI-JI goose [16]	Round or oval				Many; diameter 285 nm	Plenty of mitochondria and rough endoplasmic reticulum
Beagle dog [17]	Round		Round		Several; diameter 233–465 nm	Rough endoplasmic reticulum, mitochondria, dissociated ribosomes and lysosomes
Rhesus monkey [17]	Round or oval		Round		Numerous; diameter 200–450 nm	

## Supplementary Materials

Supplementary materials can be found at http://www.mdpi.com/1422-0067/17/4/543/s1.
