# Supplementary material for: The Long Intron 1 of Growth Hormone Gene from Reeves’ Turtle (Chinemys reevesii) Correlates with Negatively Regulated GH Expression in Four Cell Lines
Source: Int J Mol Sci. 2016 Apr 12;17(4):543. doi: 10.3390/ijms17040543 (PMC4848999; doi:10.3390/ijms17040543)
Supplement: Supplementary file 1 [file ijms-17-00543-s001.pdf]

Supplementary Materials: The Long Intron 1 of *Growth Hormone* Gene from Reeves' Turtle (*Chinemys reevesii*) Correlates with Negatively Regulated GH Expression in Four Cell Lines

Wen-Sheng Liu, Jing-E Ma, Wei-Xia Li, Jin-Ge Zhang, Juan Wang, Qing-Hua Nie, Feng-Fang Qiu, Mei-Xia Fang, Fang Zeng, Xing Wang, Xi-Ran Lin, Li Zhang, Shao-Hao Chen and Xi-Quan Zhang

Table S1. The first-intron length of *GH* gene in species.

Species	The Length of Intron 1 (bp)	*GH* Length (aa)	Transcript ID
Dog	222	216	ENSCAFT00000020118
Ferret	244	216	ENSMPUT00000014918
Mouse	183	216	ENSMUST00000103071
Rat	185	216	ENSRNOT00000015818
Pig	244	217	ENSSSCT00000018816
Guinea Pig	235	216	ENSCPOT00000022548
Marmoset	244	217	ENSCJAT00000038267
Chimpanzee	265	168	ENSPTRG00000030805
Human	272	217	ENSG00000136487
Cow	248	217	ENSBTAT00000022885
Xenopus	632	208	ENSXETG00000003984
Tetraodon	367	78	ENSTNIT00000004309
Zebrafish	199	210	ENSDART00000055675
Elephant	215	216	ENSLAFT00000020859
Cat	215	216	ENSFCAT00000003503
Horse	213	216	ENSECAT00000009392
Chicken	915	216	ENSGALG00000000249
Duck	1500	212	ENSAPLT00000004693
Tilapia	96	207	ENSONIT00000011566
Poeciliareticulata	88	204	NC_024338.1
Stickleback	505	203	ENSGACT00000019617
Coelacanth	4267	218	ENSLACT00000001338
Rabbit	188	187	ENSOCUT00000004342
Painted turtle	1214	187	NW_007281340.1
Green sea turtle	4816	217	NW_006675160.1
Turtle	2486	217	EF424785

Table S2. Detailed information on primers of the turtle *GH* gene.

Primer Pair		Base Composition (5′–3′)	Length (bp)	Amplified Region	Product Length (bp)	Annealing Temperature (°C)
GP1	F	ggcacctcagac	12	nt454-624 [a]	171	46
	R	gccgtagttctt	12			
GP2	F	gcccagcagaaatcagacat	20	nt343-636	294	63
	R	gcaggacaacaggccgtag	19			
GP3	F	aacagagagaagatgacacagat	23	nt411-678	268	63
	R	ctgtggtggtgaagctgtag	20			
TGSP1		cttcgttccggagattgatgtca	23	nt1-66 [a]	66	61
TGSP2		aagcctccggggatttcaagtc	22	nt721-792 [a]	72	61
GIP1	F	aaacatccagcttcaaacag	20	In-1	2486	61.6
	R	gcattggcaaacagact	17			
GIP2	F	gcccctctccagtctgtttgc	21	In-2	1219	58
	R	gggcatcatcttttcctgtgg	21			
GIP3	F	tcagcattttgttattcagaa	21	In-3 [b]	890	53
	R	agtccctcagtttttcataga	21			
GIP4	F	tttggcacctcagacaga	18	In-4	3131	62.6
	R	ggtgcagttgctctctccgaag	22			

[a] Based on the full-length cDNA of the turtle *GH* gene sequence we obtained (GenBank accession number: EF424785); [b] Based on the β-*actin* gene of chicken (GenBank accession number: L08165.1) bp, base pair; F, factor; R, receptor.

Table S3. Primer pairs information of turtle *GH* gene for plasmid construction.

ID	Prime Name	Sequence (5′→3′)	Restriction Site
T1	tGH-DNA-F	<u>CTAGCTAG</u>CATGGCTTCAGGTACCT	Nhe◎
T2	tGH-cDNA-F	<u>CAAGCTTG</u>ATGGCTTCAGGGTCATAT	HindIII
T3	tGH-R-6H	<u>GGAATTCC</u>GTCA*atggtgatggtgatggtg* AATGGTGCAGTTGCT	EcoR◎
T4	tGH-exon2-2675-F	CAGTCTGTTTGCCAATGCCGTG	–
T5	tGH-exon2-2675-R	CACGGCATTGGCAAACAGACTG	–

Italic bases in low case were His-tag sequence, sequence with underline was protective bases and enzyme loci.

Table S4. Sequence information of turtle *GH* gene.

Sequence Name	Temple	Primers	Length
tGH-6H	cDNA	T2 T3	688 bp
tGH-E1-IN1-E2	DNA	T1 T5	2617 bp
tGH-E2-E5	cDNA	T3 T4	553 bp
tGH-in-6H	tGH-E1-IN1-E2, tGH-E2-E5	T1 T3	3158 bp

Table S5. Primers for PCR.

Name		Primer Sequence (5′→3′)	Accession N. in GenBank	Product Size (bp)
tGH	F	CACCTGCTGGCTGCTGACACAT	EF424785	140
	R	TGGGCATCATCTTTTCCTGTGG		
DF-1	F	TCATTGTGCTAGGTGCCA	L08165	197
	R	CCTCTTCCAGCCATCTTT		
CHO	F	TGTCACCAACTGGGACGATA	NM_001135968.1	139
	R	CTGGGTCATCTTTTCACGGT		
293T	F	GGCCAACCGCGAGAAGA	U20114	164
	R	CCTCGTAGATGGGCACAGTGT		
Neo	F	ACAGACAATCGGCTGCTC	EF550208.1	186
	R	CCCGCTTCAGTGACAACG		
tGH1	F	GAATTCAAGCTTATGGCTTCAG	EF424785	161 & 2647
	R	GTGTCAGCAGCCAGCAGGT		
tGH2	F	TGGCTTCAGGGTCATATTT	EF424785	125
	R	CAGCACGGCATTGGCAAAC		

F, forward primer; R, reverse primer.

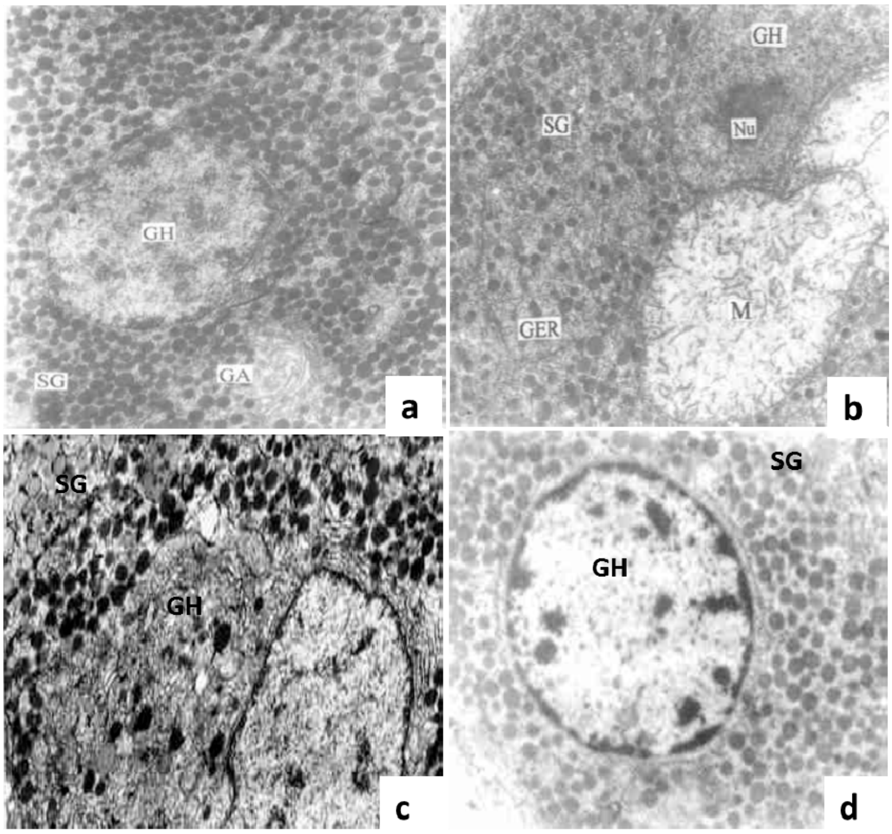

Figure S1. The ultrastructure of GH cell in *Oreochromis nilotica* during the winter (**a**) and reproductive season (**b**) [15]; The ultrastructure of pituitary *GH* cells in *Silurus meridionalis* (**c**) [19] and in Beagle dog (**d**) [17]. GH: growth hormone cell; SG: secretory granules; GA: Golgiosome body; Nu: nucleolus; GER: endoplasmic reticulum; M: Mitochodria.

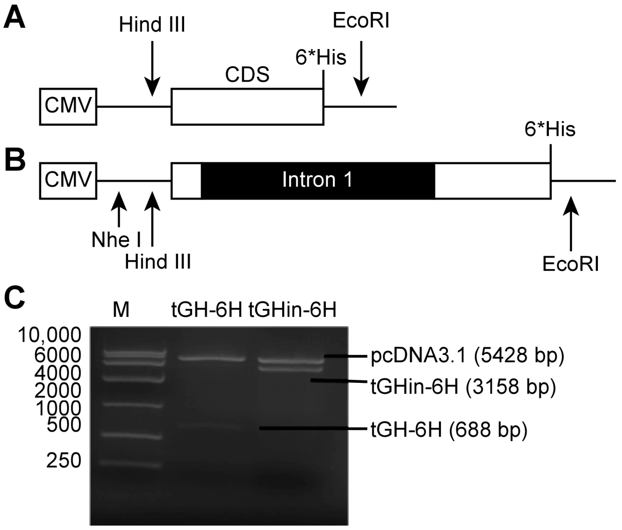

Figure S2. Diagrammatic representation of the vectors used in this study. (**A**) pcDNA3.1tGH-6H; (**B**) pcDNA3.1tGH-in-6H; (**C**) pcDNA3.1tGH-6H plasmid identification via gel electrophoresis after digestion by Hind III and EcoRI and pcDNA3.1tGH-in-6H plasmid identification after digestion by NheI and EcoRI. M represents the marker used. tGH-6H and tGHin-6H respectively represent the products created following double digestion of pcDNA3.1tGH-6H and pcDNA3.1tGH-in-6H.

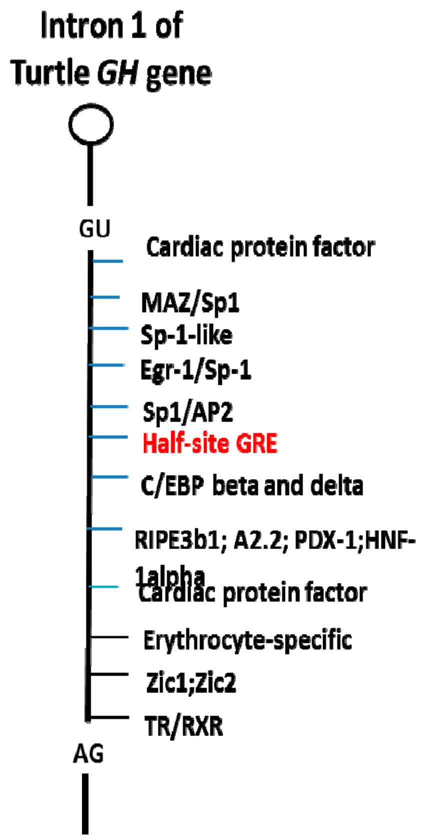

Figure S3. Several different binding sites of transcriptional elements were found among the intron 1 sequence of the turtle *GH* gene. Softberry was used to predict binding sites oftranscription factors. Some of which were in sense strand (**blue** line), while some were anti-sense strand (**black** line). GRE (GC response element) half-site (TGTTCT) was found in the first intron of turtle *GH* sequence, what is worthy studying is that these sites may have important functions in the expression of *tGH* gene.

AAACATCCAGCTTCAAACAGCACCTGAGCAACTGCAACTTACCAAAGAGAAATGGCTTCAGgtaccttttatatc
tgaattgtaaagtactgtgaacatcagtgcaggctcaggatagtgggacgcagtgcagagaaaggacagtattgccgtgtgcagtgtaaact
caatgtctgtggtaaaagatgggacattcaaacccgataaaggaaatatgttttcacacaatgcgtaattagcttgtggaactcactgccaca
ggaagatgtggaggccaagaatttagcaattttaaaaggggattggacatttctatggacacaggaatatccagagttataacctctattatg
aaggcatattaaatctgctgctccagggcttaagccactctctaactattagaggccaggacctatatgtgagggcagattatttcatgcctgc
taatgcagggtccttgcaccttcctctgaagcagctggtgctgggcagtgttagagccaggatactggactagaggggcctcaggtctgatcc
agcctggtgatttctgtgttcctaagcaacagggaggttatcattttaatcacacaaaagttgggaagttctaagttatggtttttaaagctcag
ctccattgcactaatcctaagggtggccaatgtagaggggggttgtaagcttgttggggcagggatctctgctgtgtttgtacagtgcctagcac
aaggaggtcctggaccatgacagataataaaacagctggtttgctggggcatgtgggtatgttcagtggtgctggagcaatagggcatagct
gccccatctagcacatccacacccctgctgcaaaggagccttgctccacccctcactgatccccagccctgcagctgcaggaggtcagcatc
acctgggataaatactgcctggctccatggtagtctggcaggtggggtgaggaggctctcttcccttcctcagctcctgggggcgggggtgct
ctcattcaaggaaggggagtgaaaggggaagggggagggagactgcagaagactgagactcatttgctccactcctctctctcaccttaaa
ctcatcttcaggctcctaagggttaaaccctgcttcacatgtgccaagagagcaaacctggcagtcttggaagagacactcgagtgggtgttt
tccccttttcattagcaacacgtatttactccgattccaagtgttctttgttctgagctcagtgacaccttgtgttcattaaggttcaggatataatc
tgctggtctggctcagcccttagctgtgccctgcctcaccccggcttttcaagttttagctcctttctcaagccatatacactttgtctgggctggg
gacctctgcgttcagatgccggctgatctcagatacagggagagttgccggctttccgtggcactgcattgctctgatcagcagagtagctcag
cctcttgggatgctgagatgcctctctgggaaatggtttgtgacggtctgtggttttgtctggtttgaagagcctgtgacagtttcaaaggtatg
actgacagacactaatgtgatccccccttgggaaagctcagcagtagtggcagagaaggtgctctatggtggttcacaagatatcagaactc
aggcatcccgttattctgactatgcctttatcactccacttcagttctgagctgcttttcgaggctcctctctcaagacctcagggctaaaaacaa
tgcctcatgcaacctggcagactggaatcccagggtacaaagcagcatggacaggttttggggatggaaacgaaggctacaaataactgc
ctgctatgttgctcctgcagttatttgcacacgatttagatcacccagacgtctatggccataatacgtggaataatcaatgactcaggtgtcta
agtaacacaaactgtgagggcaaaattgaccggcctctcagtgaaagactcagcctgttaattgtccaagagaaagctaaaaaggtgactt
gatcctgatctgtaaggacctccaggaggtgaagatttctgatcatagagggctctttaatccagcagacaaggcagaacaagagccaatgg
atggaagctgaagctggaataaggagcgcatttgtaacggcaagggtaattaatcatgggaacagcttattcagggacatgggggattctc
cggcacttgctgtcagtcaggactggatgtcttttggagagatactctcgagctcaactggaagttctgggtgtgatgcaggttgagattgtct
ggcctgtgctatacaggaggtgaaaaaagataatcaggatggtctataatgtatgaagcagtcaccagctgctcctttgaacccccccccc
atccgctttccctgtgtgccagccctggaaagagcaaaggggatggaaggggcttcattgtagcctcccccacacccacccccaacagtaat
taggccaccgcccccaggattactgacaccgcccctttcttttgcagGGTCATATTTTTCTCCTCTGCTCTTTGTTGCAATCATC
CTGGGGCTGCAGTGGCCACGGGGGGCAGCAGCCTTCCCTGCCATGCCCCTCTCCAGTCTGTTTGCCAATGC

Figure S4. Sequencing result of intron 1 from *C. reevesii*'s *GH* gene. Small letters were introns. Shadow letters were exton. Capital letters underline were binding sites of the primers of front and back.
